# Two-Stage Total Laparoscopic Liver Resection at a Tertiary Center in Saudi Arabia: A Report of Two Cases

**DOI:** 10.7759/cureus.76884

**Published:** 2025-01-03

**Authors:** Mohammed F Alsunaidi, Lamia S Alsubaie, Rafif E Mattar, Faisal A Al-alem

**Affiliations:** 1 Department of Surgery, King Saud University, College of Medicine, Riyadh, SAU

**Keywords:** hepatectomy, laparoscopic, liver, metastasis, minimally invasive, two stage

## Abstract

Laparoscopic liver resection (LLR) is a minimally invasive surgical approach. Initially utilized for low-risk procedures, such as the resection of benign lesions, now LLR has evolved to include more complex operations such as metastatic lesions. We present in this article two cases with liver metastasis who underwent a successful two-stage total LLR: a 57-year-old man diagnosed with sigmoid cancer and liver metastasis and a 36-year-old man diagnosed with pancreatic neuroendocrine tumor and liver metastasis.

## Introduction

When laparoscopic liver resection (LLR) first began, it was only utilized for low-risk procedures, such as minor resections and peripheral lesions. LLR is currently widely used to do minor and major resections [1]. Even more sophisticated LLR are now feasible [2]. A quick adoption of a new surgical method without adequate training might result in unanticipated difficulties, as the history of minimally invasive surgery shows [3]. The indications for LLR are growing as surgeons acquire more skills and LLR becomes more popular [1]. Nowadays, LLR has gained popularity, and it has a short-term advantage over open liver resection (OLR), as well as no less prosperous results in terms of oncology and survival rate than open surgery [4]. The two-stage liver resection procedure has commonly been done via an open technique, due to its complexity. More recently, laparoscopy has been introduced into major hepatic surgery, to allow a faster patient recovery, to decrease the use of analgesics and pain postoperatively, as well as for a smaller wound in comparison to the open technique [5]. According to studies, LLR reduces the incidence of portal hypertension, ascites, and postoperative hemorrhage. It also lowers insensible fluid loss since the viscera is not exposed. As a result, the requirement for intravenous fluid is minimized, and the third spacing that leads to hyperaldosteronism is avoided [6]. We outline a couple of instances of two-stage LLR in a tertiary hospital in Saudi Arabia.

## Case presentation

Case 1

**Figure 1:**
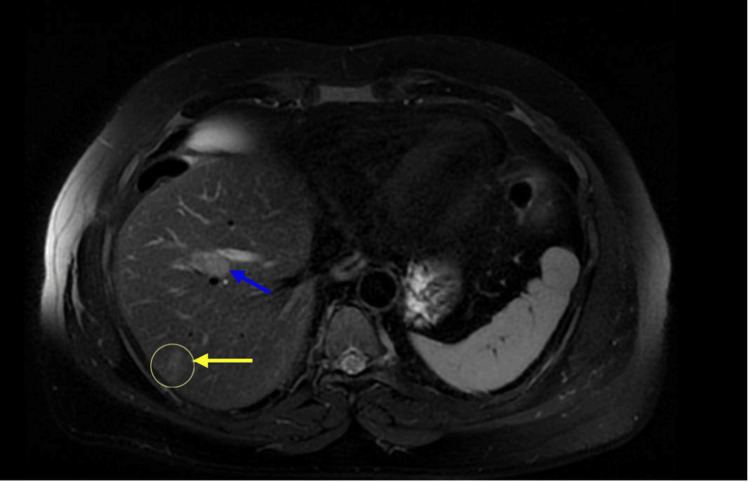
Abdominal MRI showing multiple liver metastatic lesions: blue arrow showing a lesion on the right pedicle and yellow arrow showing a lesion in segment 6 MRI: magnetic resonance imaging

**Figure 2:**
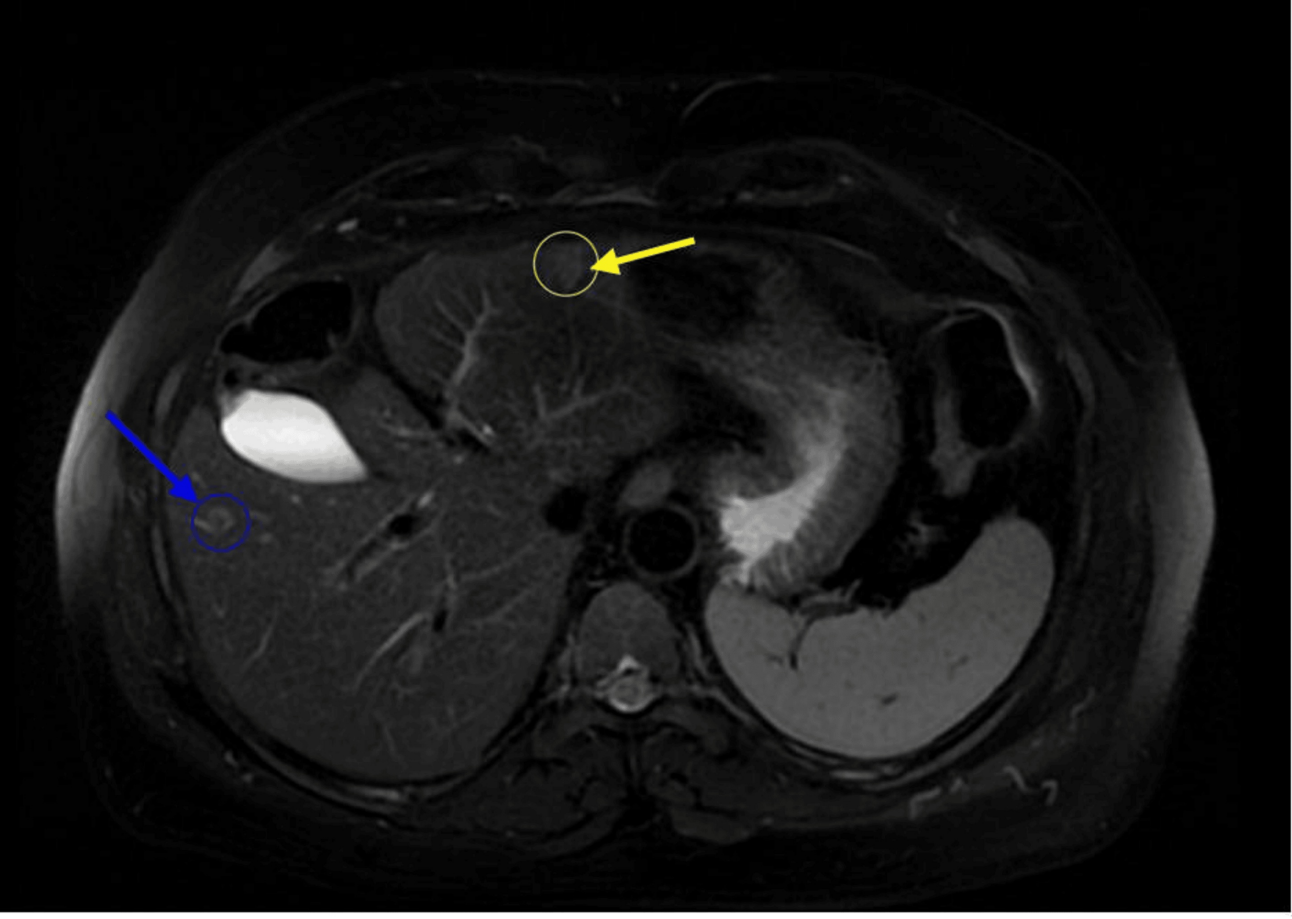
Abdominal MRI showing multiple liver lesions: yellow arrow showing a lesion in segment 3 and blue arrow showing a lesion in segment 5 MRI: magnetic resonance imaging

Our first patient is a 57-year-old gentleman diagnosed with sigmoid cancer and metachronous liver metastasis. He underwent a laparoscopic anterior resection, followed by seven cycles of adjuvant XELOX chemotherapy. One year and three months later, computed tomography (CT) showed three hepatic lesions. Magnetic resonance imaging (MRI) confirmed multiple right lobe and two left lobe metastatic lesions (Figure 1, Figure 2).

He was planned for a two-stage liver resection, aiming to clear the left liver, followed by chemotherapy and reassessment afterward for a possible right liver resection according to the chemo response.

In the first stage of resection, a total of four ports were used: Hasson's technique 12 mm infraumbilical port, 11 mm subxiphoid port, and two 12 mm ports in the left lateral and right lateral. Intraoperative liver ultrasound showed five lesions in laparoscopic wedges performed of segments 2, 3, and 4b, as well as right portal vein ligation and cholecystectomy. A drain was inserted in Morrison's pouch.

The patient tolerated the procedure well and was discharged home on the fourth day post-op; however, he was readmitted on day 8 due to an abdominal collection. This was managed by CT-guided drainage, and he was discharged home eight days later doing well. Twenty days following discharge, the patient was started on chemotherapy. He received five cycles of FOLFOX/cetuximab. Following chemotherapy, MRI showed a decrease in the size of the residual metastatic segment 8 lesions, with no new focal hepatic lesions. Two months post-op, CT done showed an interval increase in the volume of the left hepatic lobe, and restaging showed disease control. Twenty-two days after the completion of chemotherapy, the patient was admitted for the second stage of liver resection. A total of five ports were used: Hasson's technique 12 mm infraumbilical port, 12 mm subxiphoid port, 12 mm supraumbilical port, and two 5 mm ports in the left upper quadrant (LUQ) and right upper quadrant (RUQ). After adhesions were taken down, a right hepatectomy was performed. Pathology confirmed negative margins. A drain was placed at the subhepatic space.

The patient's postoperative course was unremarkable and he was discharged home on day 6 post-op. Around 27 days later, he returned to chemotherapy and completed 13 cycles. We have followed the patient for 36 months, and till the present day, he remains disease-free and in good health.

Case 2

**Figure 3:**
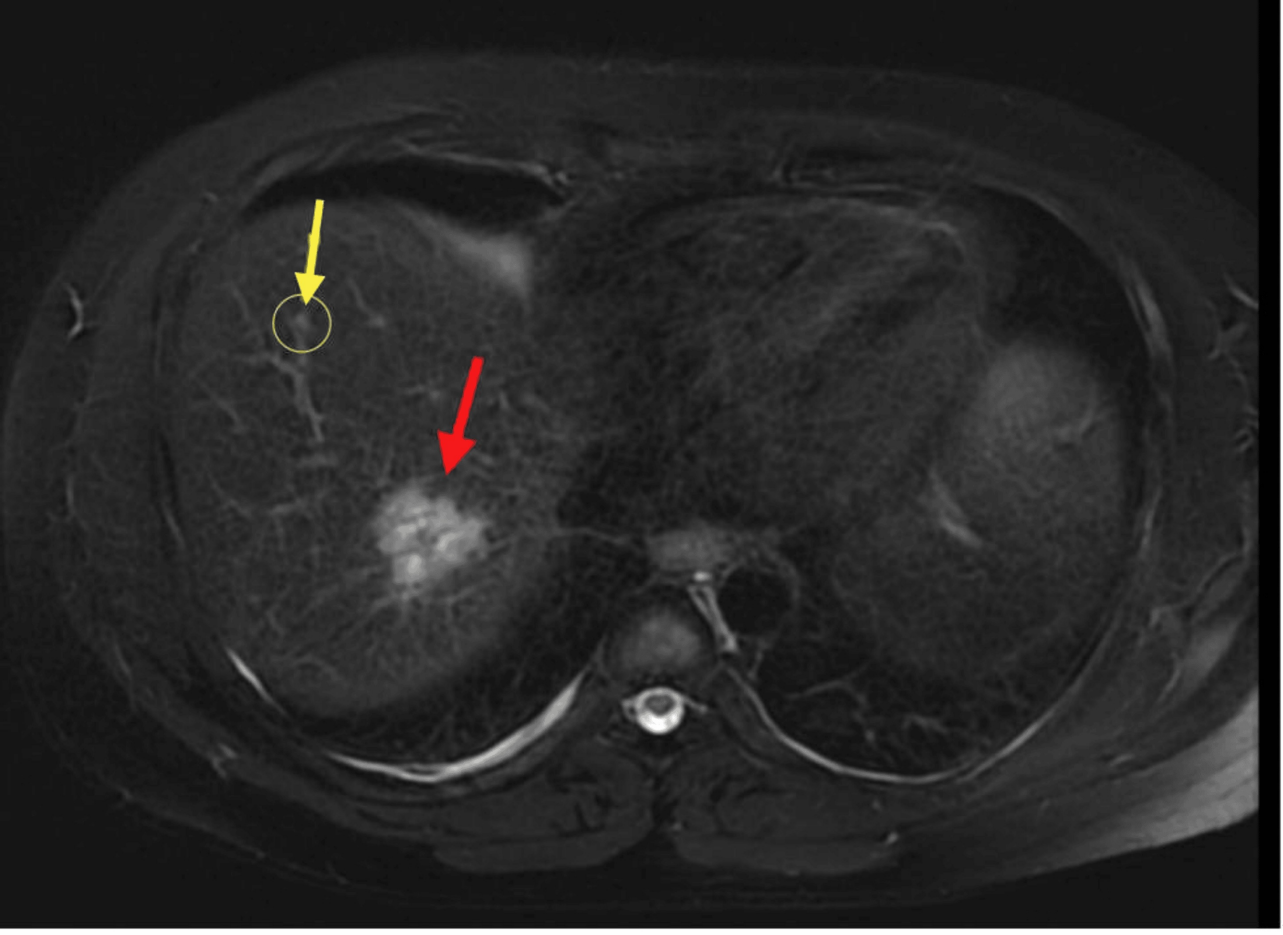
Abdominal MRI showing multiple liver lesions: yellow arrow showing a lesion in segment 8 and red arrow showing a lesion in segment 7 MRI: magnetic resonance imaging

**Figure 4:**
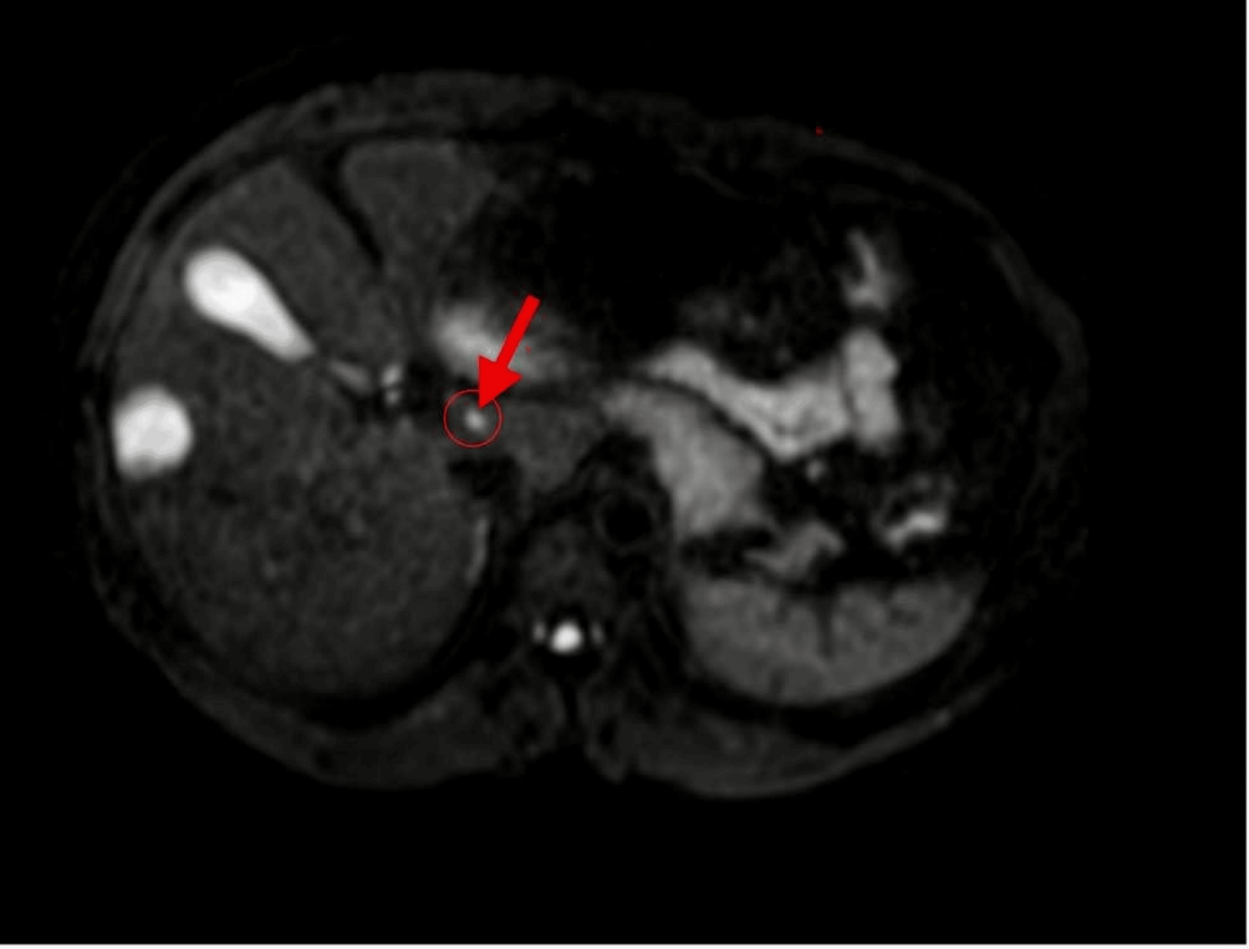
Abdominal MRI showing a lesion in the caudate lobe MRI: magnetic resonance imaging

Our second patient is a 36-year-old gentleman diagnosed with pancreatic gastrinoma and liver metastasis. He initially presented with an upper gastrointestinal bleed. Esophagogastroduodenoscopy (EGD) done showed narrowing and ulcers in the duodenum at D1 and D2 junctions. CT showed a focal hypervascular pancreatic head mass with focal areas of necrosis, invading the pylorus with no sign of obstruction. The mass was circumferentially encasing the gastroduodenal artery (GDA), with no direct invasion or tumor thrombus. The hepatic artery was spared. The portal confluence and superior mesenteric vein (SMV) were also invaded. There were also multiple hypervascular arterially enhancing focal hepatic lesions involving almost all hepatic segments, with washout in subsequent phases. Some of the lesions showed central necrosis. MRI confirmed these liver lesions. A liver biopsy was performed and showed possible functioning (gastrinoma). An octreotide scan confirmed an octreotide-avid pancreatic mass with multiple hepatic metastases. The patient was started on an octreotide injection, 30 mg IM monthly. After five months, follow-up CT showed an interval partial response to the treatment, with a stable hypervascular pancreatic head mass. However, two new hypervascular hepatic metastatic lesions had appeared. Thus, everolimus was added to the patient's regimen for one month. MRI showed a grossly stable spiculated pancreatic head mass involving and encasing the root of the mesentery, the posterior wall of the gastric pylorus, the medial wall of the second part of the duodenum, and the hepatic flexure of the colon (Figure 3, Figure 4).

The mass was also invading the SMV (including its first jejunal branch). The mass also invades the GDA. The liver demonstrated persistent diffuse homogeneous moderate to severe steatosis, with homogeneous contrast enhancement. The metastatic lesions involved all hepatic segments except parts of segments 3, 4b, and 1. All the lesions were located subcapsularly, except the lesion seen in segment 4. There was no new focal hepatic lesion.

Two weeks later, the patient was taken for the first stage of liver resection, aiming to clear the left liver. Five wedge resections of lesions in segments 2, 3, and 4b and caudate lobes were performed, as well as cholecystectomy. A drain was placed in the subhepatic space. 

Two days post-op, right portal vein embolization (PVE) was done. A week later, CT showed compensatory hypertrophy of the left hepatic lobe currently measuring 11 × 15 × 10 cm (craniocaudal (CC) × transverse (TR) × anteroposterior (AP)).

He was then taken for the second stage of liver resection two months afterward. A total of six ports were used: 12 mm infraumbilical port, 12 mm epigastric port, 55 mm port in the LUQ and left flank, 55 mm port in the right flank, and 15 mm port in the RUQ. The resection plane was marked via the PVE demarcation. A laparoscopic right hepatectomy was performed (Video 1). The patient tolerated the procedure well and was discharged home on the third postoperative day.

**Video 1:** Second stage of the two-stage total laparoscopic liver resection

The patient remained in good health during this time, and thus, we began planning for the resection of his primary pancreatic mass afterward.

## Discussion

In the past, bilateral liver metastasis was considered a contraindication for surgery. However, with the concept of future liver remnant (FLR) and liver augmentation, the inclusion criteria have expanded. LLR for the first-stage procedure is safe and viable. Its benefits include little postoperative pain, a short length of stay, early chemotherapy, low rates of morbidity and mortality, as well as frequent progression to the second stage without ever adversely compromising oncologic outcomes [7,8]. It was also found that LLR in treating colorectal liver metastasis exhibits lower blood loss, fewer complications after surgery, shorter hospital stays, earlier chemotherapy administration, and possibly increased overall survival by five years [5]. In addition, other studies have demonstrated the effectiveness of the strategy in colorectal liver metastasis [9-11].

As evidence for the safety and viability of the two-stage laparoscopic hepatectomy procedure, both patients tolerated the procedures very well with no postoperative complication, no blood transfusion needed, and a short length of stay.

However, we did not find any local reports with similar procedures. Even globally, this procedure is rarely performed in a laparoscopic manner. One of the reasons behind this has been explained in a meta-analysis since this procedure has been implanted very recently; the first case had been reported was only in 2016 [12]. Laparoscopic liver surgeries are not being used frequently, and one of the reasons is about learning how to do it by surgeons as this technology improved recently, yet the learning curve is steep [13]. Until now, laparoscopic liver procedures have been shown to be effective, and surgeons should be encouraged to seek training, to practice it more, and to also create new methods through it. The difficulties encountered in two-stage laparoscopic surgeries were like the ones encountered in the open counterpart, for example, the need for extensive adhesiolysis and the difficulties to navigate the liver after its regeneration and rotation. Interop ultrasound was a valuable tool in navigating the liver and ensuring its anatomy. The added benefit in our cases was the reduced midline adhesion that would ease access to the abdomen during Whipple's procedure.

## Conclusions

These two cases demonstrate the feasibility and safety of a two-stage total LLR in a tertiary center. The first case involved a 57-year-old man with sigmoid cancer and metachronous liver metastases, while the second case concerned a 36-year-old man with pancreatic gastrinoma and liver metastases. Both patients successfully underwent two-stage LLR with minimal complications and reduced hospital stays. They also benefited from early recovery and timely initiation of adjuvant therapies.

The purpose of this case report is to highlight the feasibility and the high success rate of two-stage total LLR as a minimally invasive approach for managing complex hepatic conditions such as metastatic liver lesions at a tertiary center in Saudi Arabia. These cases emphasize the advantages of LLR, including reduced postoperative complications, faster recovery, and shorter hospital stays, compared to traditional open surgeries. By sharing our experience as the first group to perform this procedure in Saudi Arabia, we aim to inspire other centers to adopt similar techniques and expand the scope of laparoscopic liver surgeries. Additionally, this case report highlights the importance of specialized training and skill acquisition for surgeons, as the steep learning curve remains a significant barrier to widespread implementation. We also hope to encourage further innovation and research in laparoscopic liver surgeries, contributing to better outcomes and the advancement of surgical practices globally.
